# Reliable Biomarker discovery from Metagenomic data via RegLRSD algorithm

**DOI:** 10.1186/s12859-017-1738-1

**Published:** 2017-07-10

**Authors:** Mustafa Alshawaqfeh, Ahmad Bashaireh, Erchin Serpedin, Jan Suchodolski

**Affiliations:** 1 Bioinformatics and Genomic Signal Processing Lab, ECEN Dept., Texas A&M University, College Station, 77843-3128 TX USA; 2 College of Veterinary Medicine and Biomedical Sciences, Gastrointestinal Laboratory, Texas A&M University, College Station, 77843-3128 TX USA

**Keywords:** Biomarker detection, Metagenomics, Matrix decomposition, Alternating direction method of multipliers, Augmented Lagrangian

## Abstract

**Background:**

Biomarker detection presents itself as a major means of translating biological data into clinical applications. Due to the recent advances in high throughput sequencing technologies, an increased number of metagenomics studies have suggested the dysbiosis in microbial communities as potential biomarker for certain diseases. The reproducibility of the results drawn from metagenomic data is crucial for clinical applications and to prevent incorrect biological conclusions. The variability in the sample size and the subjects participating in the experiments induce diversity, which may drastically change the outcome of biomarker detection algorithms. Therefore, a robust biomarker detection algorithm that ensures the consistency of the results irrespective of the natural diversity present in the samples is needed.

**Results:**

Toward this end, this paper proposes a novel Regularized Low Rank-Sparse Decomposition (RegLRSD) algorithm. RegLRSD models the bacterial abundance data as a superposition between a sparse matrix and a low-rank matrix, which account for the differentially and non-differentially abundant microbes, respectively. Hence, the biomarker detection problem is cast as a matrix decomposition problem. In order to yield more consistent and solid biological conclusions, RegLRSD incorporates the prior knowledge that the irrelevant microbes do not exhibit significant variation between samples belonging to different phenotypes. Moreover, an efficient algorithm to extract the sparse matrix is proposed. Comprehensive comparisons of RegLRSD with the state-of-the-art algorithms on three realistic datasets are presented. The obtained results demonstrate that RegLRSD consistently outperforms the other algorithms in terms of reproducibility performance and provides a marker list with high classification accuracy.

**Conclusions:**

The proposed RegLRSD algorithm for biomarker detection provides high reproducibility and classification accuracy performance regardless of the dataset complexity and the number of selected biomarkers. This renders RegLRSD as a reliable and powerful tool for identifying potential metagenomic biomarkers.

## Background

Thanks to the progress witnessed by the high-throughput sequencing technologies, large-scale investigation of bacterial collectivities has become possible by means of metagenomic approaches. This large-scale analysis lead to the discovery of bacterial groups that could not be analyzed through the conventional cultivation-based methods (90% of microbes are not recognized yet and not cultivable [1, 2]). In addition to bacterial composition, metagenomic techniques employed the whole-metagenome shotgun sequencing methods to infer the functional role of microbial colonies [3, 4].

Recently, several metagenomic studies have pointed out that the distortion of the normbiosis state of bacterial communities is a key player in the progression of many diseases such as obesity [5–7], diabetes [8], inflammatory bowel disease (IBD) [9], and cancer [10, 11]. These findings suggest employing microbes as possible biomarkers for the health status and certain diseases of the host. Currently, the determination of microbial biomarkers is carried out by finding the operational taxonomic units (OTUs), whose corresponding abundances differentiate for samples pertaining to distinct phenotypes.

Biomarker detection is crucial to understand disease development and design antibiotic and/or probiotic therapies. Mathematically, the task of biomarker identification can be formulated as determining the most revealing features that can differentiate multiple sets of samples or conditions (i.e., various stages of a disease, different categories of diseases, etc.). The methods proposed in literature to address the biomarker discovery problem can be classified into two categories: machine learning (pattern recognition) methods and statistical methods, respectively.

In general, the statistical approaches tackle the problem by using a statistical hypothesis test to calculate the statistical significance (i.e., p-value) of each feature. Then, the features associated with p-values lower than a well-selected level are declared as potential biomarkers. A major issue linked with the statistical-based methods is the multiple comparisons problem, which is commonly solved by substituting the p-values with the corresponding false discovery rates (FDRs). Metastats [12] and LEfSe [13] are the current standard approaches that belong to this category. Specifically, Metastats utilizes the permutation t-test and the exact Fisher’s test for non-sparse and sparse features, respectively [12]. On the other hand, to improve the robustness of biomarker discovery, LEFSe relates the statistical study with the impact of size estimation [13]. In particular, LEFSe exploits the Kruskal-Wallis and Wilcoxon-Mann-Whitney detection algorithms for class and subclass comparative studies, respectively.

In the machine learning framework, the problem of detecting the biomarkers is formulated as a feature determination task. The filtering methods are the most widely adopted approaches for biomarker detection. In filtering methods, each OTU is assigned a score based on the relevance between its abundance levels across the samples and the class labels of the samples. The operational taxonomic units that present the largest scores are declared as potential biomarkers. This scoring process is carried out one by one for each OTU and separately of the other OTUs. Therefore, filtering methods are computationally fast and easily interpretable. However, the individual ranking ignores the inter-dependencies among different variables.

Contrary to the individual ranking, the feature transformation-based methods try to generate more revealing features where each newly detected feature is depedent of all the original features. Considering all the initial characteristics in the construction of new traits accounts for the interactions between OTUs. Transformation approaches are divided broadly into two categories based on whether the labels of the samples are considered in the transformation process. These categories are the supervised and unsupervised approaches. Linear discriminant analysis (LDA) and partial least-squares (PLS) represent the two most employed supervised approaches. On the other hand, the principal component analysis (PCA) presents itself as one of the most remarkable unsupervised methods.

Identifying the most discriminating features in metagenomic datasets is a challenging task. One major challenge is that the number of biomarkers might be much larger than the number of available samples, a condition that it is commonly termed as the ‘high dimension low-sample size (HDLSS)’ problem. The HDLSS problem is also associated with serious analytical challenges [14, 15]. In addition, metagenomic analysis presents its own challenges such as: (i) metagenomic-specific artifacts such as sequencing errors and chimeric reads [16, 17], (ii) high dynamics of the bacterial populations due to the complex interactions with the host [18] and between its members [19–21], and (iii) inter-subject variability. For example, the results of [6] show that the gut microbiota of twins differ significantly.

These challenges point to a severe inconsistency issue that blocks the current biomarker identification methods from selecting the true biomarkers. For example, the authors of [22] reported that out of the 70 genes that were suggested as potential biomarkers for breast cancer by the two gene expression studies [23, 24], only three genes were found to be common. Therefore, developing a robust biomarker detection algorithm that ensures the reproducibility of the outcomes obtained from biological data plays a critical role in infering correct biological statements and making use of these results in good clinical decisions.

Toward this end, we propose herein paper the Regularized Low Rank-Sparse Decomposition (RegLRSD) algorithm for biomarker detection. RegLRSD formulates the biomarker discovery problem as a matrix decomposition problem and provides an efficient solution for this decomposition. In particular, RegLRSD models the bacterial abundance data as the superposition of a sparse matrix and a low-rank matrix. The motivation for this is due to the fact that most of microbes do not play any role. Hence, the abundance profiles of these uninformative bacteria do not vary between samples associated with different phenotypes. Therefore, considering their abundance profile as a low-rank matrix is natural. In addition, few microbes may be relevant to the biological condition under study. Consequently, the abundance profiles of these relevant microbes are expected to vary significantly between the different phenotypes. Therefore, modeling these informative bacteria as a sparse matrix is legitimate.

To improve the accuracy of extracting the low-rank and sparse matrices, we exploit the prior knowledge that the abundance profiles of non-informative bacteria do not exhibit significant variation. This is achieved by adding a smoothness constraint on the recovered low rank matrix. The RegLRSD algorithm presents several advantages. First, RegLRSD improves the reproducibility performance because of the following traits: (i) RegLRSD incorporates prior knowledge in the detection process, which constrains the analysis. Consequently, this mitigates the conventional challenges associated with the HDLSS nature of metagenomic data. (ii) The multivariate nature of RegLRSD algorithm accounts for the complex interactions between the members of the bacterial community. This contrasts the univariate-based methods (i.e., statistical hypothesis testing and filtering techniques) that ignore such sophisticated relationships between bacteria. Second, the proposed matrix decomposition formulation is convex. This provides several benefits such as: (i) global optimality, (ii) efficient solvers, and (iii) flexibility to add convex constraints without affecting the convex structure of the problem. Third, unlike feature transformation-based algorithms, the output of RegLRSD is easily interpretable in the sense that it keeps the features in their original domain.

This paper also sheds light into the design of an evaluation protocol which provides a fair and an accurate assessment of the efficiency of a biomarker detection algorithm. The absence of the “ground truth” (i.e., no absolute knowledge of the true biomarkers) prevents the objective evaluation of the biomarker detection methods. Therefore, the assessment criteria and comparisons have to be conducted with great care to make sure that all the existing prior knowledge about the true markers is taken into account.

## Methods

### Low rank-sparse model of metagenomic data

Consider the matrix **D**∈ℜ^*p*×*n*^ of bacterial abundance data, each line of **D** denotes the relative abundance of an OTU in all the *n* samples, and each column stands for the abundance values of all the *p* OTUs in one sample. In general, *p*≫*n*. Therefore, it represent a challenging high-dimensional small-sample size problem. The backbone of our approach is to capture the differentially and non-differentially abundant OTUs via a sparse matrix and low-rank matrix, respectively. In particular, most of the bacterial groups do not play any role in the considered biological system. Thus, these inappropriate OTUs are expected to exhibit high abundance levels that do not change significantly between two different phenotypes. Therefore, it makes perfect sense to model their abundance-level matrix as a low-rank matrix (represented by matrix **L**). Also, the abundance levels of the few key OTUs might present relevant changes between the two phenotypes. Such a condition will be captured by means of a sparse matrix (in our case, the matrix **S**). Mathematically,

1 $$ \mathbf{D} = \mathbf{L} + \mathbf{S}.  $$

### Extracting the sparse matrix via RegLRSD

Exploiting the low rank-sparse decomposition model of the bacterial abundance profiles (1), identifying potential biomarkers boils down to a matrix decomposition problem, with the aim of extracting the sparse matrix. This decomposition can be cast mathematically as the following optimization:

2 $$ \begin{aligned} &\text{minimize} ~~ \text{rank}(\mathbf{L}) + \lambda \|\mathbf{S}\|_{0} \\ &\textrm{subject to} ~~ \mathbf{D} = \mathbf{L} + \mathbf{S}, \end{aligned}  $$

where ∥**S**∥_0_ denotes the *l*_0_-norm of the matrix **S**, which by definition is equal to the number of nonzero elements in **S**. Problem (2) is commonly known as the robust PCA (RPCA) problem. This formulation of RPCA, given by (2), is highly non-convex because of the combinatorial optimization required by the rank operator and the *l*_0_-norm. However, the authors in [25, 26] pointed out that under general conditions, one *exactly* estimate both components (i.e., low rank and sparse matrices) by carrying out a convex optimization, referred to as the Principal Component Pursuit (PCP). This convex formulation is based on recent theories and results that show: (i) the *l*_1_ norm represents the closest convex approximation of the *l*_0_-norm, and minimizing *l*_1_-norm yields the sparsest solution to underdetermined linear systems [27], (ii) the nuclear norm provides a tight approximation of the matrix rank operator and minimizing the nuclear norm provides the lowest rank solution under wide assumptions [28]. Mathematically, PCP is expressed as

3 $$ \begin{aligned} &\text{minimize} ~~ \|\mathbf{L}\|_{*} + \lambda \|\mathbf{S}\|_{1} \\ &\textrm{subject to} ~~ \mathbf{D} = \mathbf{L} + \mathbf{S}, \end{aligned}  $$

where *λ* represents a positive regularization factor that monitors the degree of sparseness and smoothness in **S** and **L**, respectively. Variable ∥**L**∥_∗_ stands for the nuclear norm of **L** and is equal to the sum of the singular values. Finally, the notation ∥**S**∥_1_ denotes the *l*_1_ norm of **S**, and it is defined as the summation of the absolute values of the matrix elements.

In an attempt to enhance the estimation accuracy of **S** and **L**, we extend the formulation in (3) by adding a penalty term in order to enforce the smoothness of each row of **L**. This penalty term incorporates the prior knowledge that the abundance profiles of non differentially abundant OTUs are smooth. In this paper, the first order difference (FOD) is adopted as a measure of smoothness, which is defined as:

4 $$ \|\mathbf{X}\|_{FOD} = \sum\limits_{j}{\|\mathbf{F}\mathbf{x}_{j}\|_{1}},  $$

where **x**_*j*_ denotes the *j* ^*th*^ column of **X**, and **F** represents the first order difference operator defined as:

5 $$ \mathbf{F} =\left[ \begin{array}{cccccc} -1 & 1 & 0 & 0 &\dots & 0 \\ 0 & -1 & 1 & 0 &\dots & 0\\ \vdots & \vdots & \vdots & \vdots &\ddots & \vdots \\ 0 & 0 & 0 & \dots &-1 & 1 \end{array} \right].  $$

Thus, the RegLRSD algorithm aims to untie the optimization problem:

6 $$ \begin{aligned} (\mathbf{L}^{*},\mathbf{S}^{*}) &= \text{arg}\min_{\mathbf{L},\mathbf{S}} \left\{\vphantom{\sum\limits_{i=1}^{p}} f(\mathbf{D},\mathbf{L},\mathbf{S}) = \frac{1}{2}\|\mathbf{D} - \mathbf{L}-\mathbf{S}\|_{F}^{2}\right. \\ &\quad +\left. \alpha \|\mathbf{L}\|_{*} +\lambda \|\mathbf{S}\|_{1} + \beta \sum\limits_{i=1}^{p}\|\mathbf{F}{\mathbf{l}_{i}^{T}\|_{1}}\right\}, \end{aligned}  $$

where $\mathbf {l}_{i}^{T}$ stands for the *i* ^*th*^ row of **L**. One key advantage of this formulation is that that the optimization problem (6) is convex. The above-mentioned convex optimization formulation yields several benefits: (i) it enables a global optimal solution, (ii) it enables utilizing the well-established theory and tools for solving convex optimization problems, and (iii) it allows the luxury to take into account extra convex constraints to capture better the existing prior information. However, direct application of generic convex solvers may not be feasible due to the high dimensional nature of our problem. For example, interior point methods exhibit high order complexity. Moreover, there is no approach available to determine the jointly optimal solution for the optimization (6). Therefore, herein paper we consider an efficient alternating-based algorithm to carry out (6). The alternating-minimization approach first optimizes *f*(**L**,**S**) with respect to **S** (matrix **L** is considered constant), and then it optimizes *f*(**L**,**S**) with respect to **L** (matrix **S** being considered a fixed constant). In particular, it adopts the following updating steps:

7 $$\begin{array}{*{20}l} \mathbf{S}^{(k)} &= \text{arg}\min_{\mathbf{S}}~f(\mathbf{L}^{(k-1)},\mathbf{S})  \end{array} $$

8 $$\begin{array}{*{20}l} \mathbf{L}^{(k)} &= \text{arg}\min_{\mathbf{L}}~f(\mathbf{L},\mathbf{S}^{(k)}).  \end{array} $$

This strategy utilizes the fact that the two sub-problems (7) and (8) admit efficient solutions. In particular, the problem in (7) can be reformulated as follows:

9 $$ \mathbf{S}^{(k)} = \text{arg}\min_{\mathbf{S}} ~~ \frac{1}{2}\|\mathbf{D} - \mathbf{L}^{(k-1)}-\mathbf{S}\|_{F}^{2} +\lambda \|\mathbf{S}\|_{1}.  $$

Problem (9) admits the following closed form solution:

10 $$ \mathbf{S}^{(k)} = \mathcal{S}_{\lambda}(\mathbf{D} - \mathbf{L}^{(k-1)}),  $$

where $\mathcal {S}_{\tau }:\mathfrak {R} \to \mathfrak {R}$ denotes the *shrinkage operator*, expressed as:

11 $$ \mathcal{S}_{\tau}(x) = \text{sgn}(x) \text{max}(|x|-\tau,0),  $$

and where *τ*≥0 denotes the threshold level. In the case of a matrix, the shrinkage operator will be applied onto each constituent element of the matrix. The problem in (8) can be cast as:

12 $$ \begin{aligned} \mathbf{L}^{(k)} &= \text{arg}\min_{\mathbf{L}} \frac{1}{2}\|\mathbf{D} -\mathbf{S}^{(k)} - \mathbf{L}\|_{F}^{2} + \alpha \|\mathbf{L}\|_{*} \\ &\quad+\beta \sum\limits_{i=1}^{p}{\|\mathbf{F}\mathbf{l}_{i}^{T}\|_{1} }. \end{aligned}  $$

The current formulation of the optimization problem in (12) is neither in a format that admits a closed-form expression as (7) nor in the format of a well-established problem that admits an efficient solution. Moreover, relying on generic convex techniques to solve (12) may not be efficient. The difficulty exhibited by this minimization problem arises from the combination of the two non-smooth terms ∥**L**∥_∗_ and $\sum _{i=1}^{p}{\|\mathbf {F}\mathbf {l}_{i}^{T}\|_{1}}$. Therefore, we propose to reformulate (12) by introducing an additional variable and constraint to separate these two terms. Adding this auxiliary variable enables the decomposition of (12) into two subproblems that can be solved efficiently. The first subproblem is the *nuclear-norm regularized least-squares* (LS) optimization problem which presents a closed-form solution [29]. The second problem can be recast as the *total variation denoising* problem [30], which presents an efficient solution [31]. In particular, (12) is reformulated as:

13 $$ \begin{aligned} (\mathbf{L}, \mathbf{Y}) &= \text{arg}\min_{\mathbf{L},\mathbf{Y}} ~~ \frac{1}{2}\|\mathbf{D} -\mathbf{S}^{(k)} + \mathbf{L}\|_{F}^{2} + \alpha \|\mathbf{L}\|_{*} \\ &\quad+ \beta \sum\limits_{i=1}^{p}{\|\mathbf{F}\mathbf{y}_{i}^{T}\|_{1} },\\ \textrm{subject to}~~ \mathbf{Y} &= \mathbf{L}, \end{aligned}  $$

where $\mathbf {y}_{i}^{T}$ stands for the *i* ^*th*^ row of the auxiliary variable **Y**. To solve (13), we make use of the alternating direction method of multipliers (ADMM) [31]. In general, the ADMM algorithm converts the constrained optimization problem into an unconstrained optimization problem with a novel objective that it is referred to as the augmented Lagrangian. The augmented Lagrangian associated with the optimization (13) is:

14 $$ \begin{aligned} \mathcal{L}_{\rho}(\mathbf{L},\mathbf{Y},\mathbf{Z}) &= \frac{1}{2}\|\mathbf{D} -\mathbf{S}^{(k)} + \mathbf{L}\|_{F}^{2} + \alpha \|\mathbf{L}\|_{*} \\ &\quad+ \beta \sum\limits_{i=1}^{p}{\|\mathbf{F}\mathbf{y}_{i}^{T}\|_{1}} +\left\langle \mathbf{Z},\mathbf{L}-\mathbf{Y}\right\rangle \\ &\quad+ \frac{\rho}{2}\|\mathbf{L}-\mathbf{Y}\|_{F}^{2}, \end{aligned}  $$

where **Z** represents the Lagrange multiplier matrix. Thus, the ADMM formulation of (13) is given by:

15 $$ (\mathbf{L}, \mathbf{Y}, \mathbf{Z}) = \text{arg}\min_{\mathbf{L},\mathbf{Y},\mathbf{Z}} ~~ \mathcal{L}_{\rho}(\mathbf{L},\mathbf{Y},\mathbf{Z}).  $$

The ADMM solution of (15) is of recursive nature. Each recursion, in particular the *r*-th iteration, assumes the updates:

16 $$ \begin{aligned} \mathbf{L}^{(r)} &= \text{arg}\min_{\mathbf{L}}~ \frac{1}{2}\|\mathbf{D} -\mathbf{S}^{(k)} - \mathbf{L}\|_{F}^{2} \\ &\quad+ \alpha \|\mathbf{L}\|_{*} + \left\langle \mathbf{Z}^{(r-1)},\mathbf{L}-\mathbf{Y}^{(r-1)}\right\rangle \\ &\quad+\frac{\rho}{2}\|\mathbf{L}-\mathbf{Y}^{(r-1)}\|_{F}^{2}, \end{aligned}  $$

17 $$ \begin{aligned} \mathbf{Y}^{(r)} &= \text{arg}\min_{\mathbf{Y}}~ \left\langle \mathbf{Z}^{(r-1)},\mathbf{L}^{(r)}-\mathbf{Y}\right\rangle \\ &\quad+\frac{\rho}{2}\|\mathbf{L}^{(r)}-\mathbf{Y}\|_{F}^{2} + \beta \sum\limits_{i=1}^{p}{\|\mathbf{F}\mathbf{y}_{i}^{T}\|_{1} }, \end{aligned}  $$

18 $$ \mathbf{Z}^{(r)} = \mathbf{Z}^{(r-1)} + \rho(\mathbf{L}^{(r)} - \mathbf{Y}^{(r)})  $$

#### **Remark 1**

For any arbitrary vectors **u**,**v**∈ℜ^*n*^, and scalars *a*,*b*∈ℜ, the following relation holds:

19 $$ \left\langle a\mathbf{v}+b\mathbf{u}, \mathbf{u} \right\rangle = b\left\lVert-\frac{a}{2b}\mathbf{v} - \mathbf{u}\right\rVert_{F}^{2} - \frac{a^{2}}{4b} \left\lVert\mathbf{v}\right\rVert_{F}^{2}.  $$

Based on Remark-1, the problem in (16) is recast as:

20 $$ \begin{aligned} \mathbf{L}^{(r)} &= \text{arg}\min_{\mathbf{L}}~ \alpha \|\mathbf{L}\|_{*} \\ &\quad+ \frac{1+\rho}{2}\left\lVert\frac{\mathbf{D} -\mathbf{S}^{(k)} +\rho \mathbf{Y}^{(r-1)} - \mathbf{Z}^{(r-1)}}{1+\rho} - \mathbf{L}\right\rVert_{F}^{2} \end{aligned}  $$

According to [29], problem (20) admits the following closed form solution:

21 $$ \mathbf{L}^{(r)} = \mathcal{D}_{\frac{\alpha}{1+\rho}}\left(\frac{\mathbf{D} -\mathbf{S}^{(k)} +\rho \mathbf{Y}^{(r-1)} - \mathbf{Z}^{(r-1)}}{1+\rho}\right),  $$

where $\mathcal {D}_{\tau }$ is the *singular value shrinkage* operator defined by:

22 $$  \mathcal{D}_{\tau}(\mathbf{X}) = \mathbf{U} \mathcal{D}_{\tau}(\Sigma) \mathbf{V}^{T},~~~ \mathcal{D}_{\tau}(\Sigma) = \text{diag}(\{\sigma_{i} - \tau\}_{+})  $$

where **U**, **V**, and *σ*_*i*_ stand for the left singular vectors, right singular vectors and singular values of **X**, respectively, and the notation (*x*)_+_ denotes the positive part of *x* (i.e., (*x*)_+_= max(0,*x*)). In other words, *D*_*τ*_(**X**) employs a soft-thresholding operation onto the singular values of **X**, shifting these towards zero. This is the reason why this transformation it is also referred to as the *singular value shrinkage* operator.

Considering Remark-1, problem (17) is recast as:

23 $$ \begin{aligned} \mathbf{Y}^{(r)} &=\text{arg}\min_{\mathbf{Y}}\frac{\rho}{2}\left\lVert\frac{\mathbf{Z}^{(r-1)}+\rho \mathbf{L}^{(r)}}{\rho}-\mathbf{Y}\right\rVert_{F}^{2} \\ &\quad+ \beta \sum_{i=1}^{p}{\|\mathbf{F}\mathbf{y}_{i}^{T}\|_{1} }. \end{aligned}  $$

The rows of **Y** are updated separately according to the optimization:

24 $$ \begin{aligned} {\mathbf{y}_{i}^{T}}^{(r)} &=\text{arg}\min_{\mathbf{y}}\frac{\rho}{2}\left\|\frac{{\mathbf{z}_{i}^{T}}^{(r-1)}+\rho {\text{\l}_{i}^{T}}^{(r)}}{\rho}-\mathbf{y}\right\|_{F}^{2} \\ &\quad+ \beta {\|\mathbf{F}\mathbf{y}\|_{1} }, \end{aligned}  $$

where **z**_*i*_ and **l**_*i*_ are the *i* ^*th*^ rows of **Z** and **L**, respectively. Problem (24) is often called the total variation denoising problem [30], and it admits an efficient solution via ADMM as described in Section 6.4.1 in [31]. Alternatively, problem (24) can be cast as a special case of the Fused Lasso Signal Approximator (FLSA), which can be properly addressed via the subgradient finding algorithm (SFA) [32].

The RegLRSD algorithm is summed up via Algorithm 1.



### Extracting the differentially abundant bacteria via RegLRSD

The proposed approach for biomarkers detection assumes two stages. First, employ RegLRSD to resolve the original bacterial abundance data matrix into a low-rank matrix that models the non-differential abundant bacteria and a sparse matrix that models the differential abundant bacteria. Second, construct a scoring vector as a function of the extracted sparse matrix to rank each OTU (i.e., feature). Then, the *m* highest scores OTUs are declared as potential bacterial biomarkers.

The reasoning for employing the sparse matrix for extracting the potential biomarkers is that the abundance levels of informative OTUs can be considered to be a sparse perturbation matrix superposed over the low-rank matrix that models the abundance levels of the non-informative microbes (i.e., **D**=**L**+**S**). The stronger the variation in the abundance levels of OTUs, the larger the magnitude of the corresponding elements in the sparse matrix **S**. It is pertinent to mention that the strength of the variation of each OTU between the two phenotypes is determined by the absolute values of the non-zero entries in **S** rather than their exact values. This is because the elements of **S** could be either positive or negative based on the role (i.e., activation or deactivation) played by the microbes. Therefore, the score of the *i* ^*th*^ OTU is achieved by adding up the absolute values of the elements located on the *i* ^*th*^ line of **S**. Thus, the scoring vector **s** *v* is expressed as :

25 $$ \mathbf{v} = \left[\sum\limits_{j=1}^{n}|s_{1j}|,\hdots,\sum\limits_{j=1}^{n}|s_{pj}|\right]^{T}.  $$

### Parameter selection

RegLRSD algorithm is equipped with four regularization parameters, *α*, *β*, *λ* and *ρ* that control the impact of the rank (i.e., ∥**L**∥_∗_), smoothness $\left (i.e., \sum _{i=1}^{p}{\|\mathbf {F}\mathbf {l}_{i}^{T}\|_{1}}\right)$, sparseness (*i*.*e*.,∥**S**∥_0_), and fitness $\left (i.e., \|\mathbf {L}-\mathbf {Y}\|_{F}^{2}\right)$ penalties in (6) and (14). In order to select the appropriate values for these parameters, we relied on similar models and utilized the recommended settings proposed in literature. For example, the PCP problem (3), which is a pruned variant of the objective of RegLSRD algorithm, was addressed in [26]. In particular, PCP assumes the following objective ∥**L**∥_∗_+*λ*∥**S**∥_0_. The authors in [26] proved that under mild assumptions, the two matrices **L** and **S** can be recovered with high probability when $\nicefrac {\lambda }{\alpha } = \nicefrac {1}{\sqrt {\max \{n,p\}}}$. Therefore, in our experiments, we set *α*=1 and $\lambda = \nicefrac {1}{\sqrt {\max \{n,p\}}}$.

In what concerns the fitness penalty parameter *ρ*, which is the single parameter that is associated with the ADMM method, the ADMM technique is known for its robustness to poor selection of its parameter. Specifically, the convergence of ADMM is guaranteed, under broad assumptions, for all positive values of its parameter [33]. Here, we set *ρ*=1. In addition, herein paper, we set *β*=0.1*α*.

### Implementation and disponibility of the method

The RegLRSD algorithm is carried out in MATLAB and exploits the original codes of the SFA algorithm (i.e., "flsa" function included in the SLEP package [34]) in order to solve the subproblem (24). Therefore, RegLRSD cannot be used for commercial applications without consent from the authors of SFA algorithm and RegLRSD. To support ongoing metagenomic analysis and to extend the utility of RegLRSD for non-MATLAB users, RegLRSD is implemented as a standalone executable software package and is made available at https://sites.google.com/a/tamu.edu/mustafa/software/reglrsd. This package is provided with a graphical interface to enable the user to set the algorithm parameters and to report the detected markers.

### Nearest centroid classifier (NCC)

A nearest centroid classifier represents a special case of a distance-based supervised learning approach. The NCC-based classification approach assumes two steps. The first step trains the classifier by exploiting the labeled data (i.e., **d**_*i*_) to determine the mean (i.e., centroid) of each class. The average vakue of the *k* ^′^ *t* *h* class ($\mathbf {\mu }_{C_{k}}$) is obtained as follows:

26 $$ \mathbf{\mu}_{C_{k}} = \frac{1}{|N_{C_{k}}|}\sum\limits_{\mathbf{d}_{i} \in C_{k}}\mathbf{d}_{i}.  $$

The second step assigns a test sample (**z**) to the class that presents a closer centroid. This reduces to the optimization:

27 $$ \hat{C}(\mathbf{z}) = \text{arg}\min_{C_{k}}dis(\mu_{C_{k}},\mathbf{z}),  $$

where $dis(\mu _{C_{k}},\mathbf {z})$ stands for the distance between the test sample **z** and the centroid of the samples associated with the *k* ^′^ *t* *h* class ($\mathbf {\mu }_{C_{k}}$).

### Data description

The abundance levels of the OTUs were generated from filtered 16S rRNA gene sequencing by exploiting the naive Bayesian classifier already implemented in the Ribosomal Database Project (RDP) [35]. The reads that present confidence below 0.8 were rebinned not certain. The per-sample normalized bacterial abundance profiles were collected into a matrix, referred to as the taxonomic relative abundance matrix. RegLRSD algorithm takes this matrix as input. Due to the unsupervised nature of RegLRSD, the sample labels are not necessary.

#### Dogs with idiopathic inflammatory bowel disease (IBD) dataset

This dataset compares the fecal microbiota between 10 healthy dogs and 12 dogs diagnosed with IBD. The extracted DNA from fecal samples was sequenced by 454-pyrosequencing. OTUs were attributed by making sure at least 97% sequence similarity against the Greengenes reference database [36] using Quantitative Insights Into Microbial Ecology (QIIME) [37]. The sequencing data were stored into the National Center for Biotechnology Information (NCBI)-Sequence Read Archive (SRA) with the registration number SRP040310.

#### Dogs with exocrine pancreatic insufficiency (EPI) dataset

Three day pooled fecal samples were gathered from 18 healthy dogs and 7 dogs with EPI. Extracted DNA was sequenced by Illumina sequencer, and the generated sequences were analyzed using QIIME to obtain the final OTU table with at least 97% sequence similarity against the Greengenes reference database. The sequences can be accessed in the NCBI-SRA database under the accession number SRP091334.

#### Mouse model of ulcerative colitis (UC) dataset

This data set stands for the fecal microbiota of the mice model with UC and control mice. The description of the samples collection, processing and DNA extraction is described in [38]. The microbiota of 20 T-bet ^−/−^ x Rag2 ^−/−^ (UC) and 10 Rag2 ^−/−^ (control) mice was assessed using 16S data from fecal samples. The taxonomic relative abundance table is publicly available in the Supplementary Material of [13].

## Results and discussions

This section presents the comparison of RegLRSD algorithm with the latest existing algorithms over the three metagenomic investigations described in the Material and Methods Section. In particular, the RegLRSD algorithm is contrasted with LEFSe [13] and MetaStats [12] from the statistical biomarker detection algorithms family, MetaBoot [39] and the entropy-based filtering method from the machine learning family. Additionally, RegLRSD is compared with the RPCA algorithm for metagenomic biomarker detection [40] in order to examine the impact of adding the smoothness constraint into the original PCP problem (2).

### Evaluation criteria

The competing algorithms were evaluated based on their classification and reproducibility performance. The essence of this evaluation relies on generating a high number of variations in the original dataset. Then, the evaluation metrics are computed by averaging the results obtained over all these different variations as shown Algorithm 2. The details of the evaluation protocol is discussed in the following two subsections.



#### Reproducibility performance

The reproducibility performance of a biomarker detection algorithm is empirically measured by generating different variations of the original dataset, and comparing the output of the algorithm based on these different variations. The reasoning behind this procedure is that a stable biomarker detection approach must provide alike outcomes in the presence of small variations in the data samples. This requirement is in line with the hopes of biologists that expect that changing the sample size by taking out or including a few samples must not alter dramatically the biomarkers detected by the algorithm.

The evaluation methodology for estimating the reproducibility performance can be formalized as follows. First, divide the original dataset $\mathbf {D} \in \mathfrak {R}_{+}^{p\times n}$ into two subsets: $\mathbf {D}_{k}^{train} \in \mathfrak {R}_{+}^{p\times \left \lceil r\cdot n\right \rceil }$ and $\mathbf {D}_{k}^{test} \in \mathfrak {R}_{+}^{p\times (n-\left \lceil r\cdot n\right \rceil)}$, where *r*∈(0,1). This random division is repeated *K* times, and the sub-index *k* represents the iteration number. Second, the biomarker detection algorithm is applied on each of the *K* training subsets. This results in *K* sets of potential biomarkers (i.e., $\left \{\mathcal {F}_{k}\right \}_{k=1}^{K}$, where $\mathcal {F}_{k}$ denotes the set of identified markers when applying the algorithm over $\mathbf {D}_{k}^{train}$). Third, the pairwise similarity between the *K*(*K*−1)/2 pairs of the marker sets is measured by means of a similarity index. Fourth, the reproducibility performance of the algorithm (*C*_*avg*_) is expressed as the mean of the all pairwise similarities, i.e.,

28 $$ C_{avg} = \frac{2\sum_{i=1}^{K}{\sum_{j=i+1}^{K}{SI(\mathcal{F}_{i},\mathcal{F}_{j})}}}{K(K-1)},  $$

where *SI* stands for the similarity index that measures the similarity between any two marker sets $\mathcal {F}_{i}$ and $\mathcal {F}_{j}$. Among the variety of similarity indexes that have been proposed, the Kuncheva index (KI) [41] was adopted as a measure of similarity in this work. This is because KI includes a correction term to account for the possible bias that results from the existence of common markers among the two signature lists that are randomly selected. Formally, KI is expressed as:

29 $${} KI(\mathcal{F}_{i},\mathcal{F}_{j}) = \frac{p.|\mathcal{F}_{i} \cap \mathcal{F}_{j}| - |\mathcal{F}|^{2}}{|\mathcal{F}|~(p - |\mathcal{F}|)} = \frac{|\mathcal{F}_{i} \cap \mathcal{F}_{j}| - (|\mathcal{F}|^{2}/p)}{|\mathcal{F}| - (|\mathcal{F}|^{2}/p)}  $$

where $|\mathcal {F}|$ represents the size of the identified markers (i.e., $|\mathcal {F}| = |\mathcal {F}_{i}| = |\mathcal {F}_{j}|$). The values of Kuncheva index range from −1 to 1. Larger KI values indicate higher stability performance. Due to the correction term $(|\mathcal {F}|^{2/p})$, which accounts for selecting markers that are common among marker sets due to chance, the KI may take negative values.

In this paper, the stability performance was visualized by presenting three types of descriptive plots. The first plot shows the average KI over all pairwise comparisons. The second plot provides more details about the distribution of all the KI values by presenting their histogram. An ideal algorithm in terms of stability will have the Dirac-delta distribution at KI equal to 1. This means that the algorithm generates the same set of markers over all subsamples. Practically, the more concentrated the histogram is to the right side of the plot, the more stable is the algorithm. The third plot aims to depict the stability of the ranked microbial marker lists. This is achieved by ordering all the selected markers based on their ranks. Then, a boxplot is generated for the ranks obtained in all the *K* subsamples for each selected marker. A perfect algorithm in the sense of stability of the ranked lists will have boxplots that are centered at the 45° line, which means that the algorithm perfectly preserves the order of the detected markers in all subsamples.

#### Classification performance

Accuracy, sensitivity, and specificity are the three metrics that were used to measure the classification performance. The classification accuracy represents the fraction of the number of samples that were correctly predicted to the total number of samples. One major drawback of accuracy is that its value is dominated by the class with the majority of samples. Therefore, in case of imbalanced class distribution or when the forecast of the minority group is critical, accuracy may be misleading. Thus, class-specific measures (i.e., sensitivity and specificity) are needed to provide a more accurate picture about the classification performance. Sensitivity (specificity) is expressed as the contribution of the correct predictions in the positive (negative) class. Formally, let *TN* and *TP* represent the number of correctly identified negative and positive subjects. Consider that *FN* and *FP* represent the number of false-predicted instances in the negative and positive classes, respectively. The accuracy, sensitivity and specificity measures are expressed as:

30 $$\begin{array}{*{20}l} Accuracy &= \frac{TP+TN}{TP+FN+TN+FP}  \end{array} $$

31 $$\begin{array}{*{20}l} Sensitivity &= \frac{TP}{TP+FN}  \end{array} $$

32 $$\begin{array}{*{20}l} Specificity &= \frac{TN}{TN+FP}.  \end{array} $$

The classification performance is measured empirically according to the evaluation protocol shown in Algorithm 2. At the *k* ^*th*^ iteration, the classifier is trained by the data corresponding to the selected markers $\left (\mathbf {D}_{k}^{train}(\mathcal {F}_{k})\right)$. Then it is tested against the remaining $\mathbf {D}_{k}^{test}(\mathcal {F}_{k})$. One major benefit from repeating the evaluation *K* times is to mitigate the over-optimistic results that are associated with the conventional cross-validation on small-sample studies [42]. In our experiments, two versions of the nearest centroid classifiers were employed. The first version relies on the *l*_1_ norm, while the second version exploits the *l*_2_ norm. Therefore, herein paper, the first classifier is referred to as NCC-1, while the second one is denoted as NCC-2.

### Discussion of evaluation criteria

A critical challenge for assessing the performnce of biomarker detection approaches is the lack of information about the true biomarkers. This hampers the objective assessment of the performance of competing biomarker selection algorithms. To overcome this challenge, evaluation criteria have to be properly developed to replicate comparisons as if the true markers were known. The evaluation criteria have to capture the features of the true biomarkers. The true biomarkers exhibit two properties. The first feature is the fact that the true biomarkers must allow differentiating different phenotypes. In general, this is assessed via the performance of a classifier designed based on the selected biomarkers. The second feature relies on the fact that true signatures appear not to be sensitive against variations in the training samples. This feature is evaluated via empirical assessment of the biomarker identification algorithm stability.

A common practice is to use *only* the classification performance as a measure of the effectiveness of a biomarker detection algorithm. In addition to ignoring the reproducibility performance, relying solely on the classification performance may be misleading for several reasons. First, the classification performance depends on factors other than the quality of the selected variables (i.e., biomarkers). In particular, the preprocessing steps and classifier model employed significantly impact the classification performance. Second, in the small sample size setups, the empirical estimation of classification accuracy may not reflect the true performance of a classifier.

Unfortunately, the existing metagenomic biomarker identification schemes have not yet considered the reproducibility performance in their assessments. This calls the utility of these methods under question. Similarly, assessing a biomarker detection algorithm based on its stability performance is delusive. For example, a trivial algorithm that returns the same features irrespective of the training samples will achieve a perfect stability performance. Thus, reproducibility needs to be assessed together with the classification performance.

### Simulation setup

The classification and consistency metrics were used to measure the efficiency of the six biomarker detection algorithms in identifying potential markers. The consistency-classification evaluation protocol is presented in Algorithm 2. In our studies, a random subsampling without replacement is utilized to generate 500 subsamples (i.e., *K*=500) variations of the original dataset. Each subsample contains 80% of the samples in the original dataset (i.e., *r*=0.8). The classification and consistency performance were evaluated at different number of selected markers to provide further insights on the performance of the competing algorithms under varying sizes of the biomarker sets. The reported outcomes stand for the average over the 500 experiments.

The classification performance is measured empirically according to the evaluation protocol shown in Algorithm 2. At the *k* ^*th*^ iteration, the classifier is trained by the data corresponding to the selected markers $\left (\mathbf {D}_{k}^{train}(\mathcal {F}_{k})\right)$. Then it is tested against the remaining $\mathbf {D}_{k}^{test}(\mathcal {F}_{k})$. One major benefit from repeating the evaluation *K* times is to mitigate the over-optimistic results that are associated with the conventional cross-validation on small-sample studies [42]. In our experiments, two variants of the nearest centroid classifiers were used. The first approach employed the *l*_1_ norm as a measure of distance, while in the second approach, the *l*_2_ norm was used. In this paper, we refer to the first classifier as NCC-1 and to the second one as NCC-2.

### Discussion of evaluation criteria

A major bottleneck for the evaluation of biomarker discovery algorithms is the lack of knowledge of the true biomarkers. This hampers the objective assessment of the performance of competing biomarker selection algorithms. To overcome this challenge, evaluation criteria have to be suitably designed in order to mimic comparisons as if the true markers were known. In particular, the evaluation metrics need to capture the features of the true biomarkers. True biomarkers are characterized by two properties. The first property is that the true markers enable distinguishing between different phenotypes. Commonly, this feature is measured via the classification performance of a classifier model built using only the selected biomarkers. The second feature is that true signatures tend to be robust against the variation in the training set. This feature can be assessed through empirical estimation of the stability of the biomarker detection algorithm.

A common practice is to use *only* the classification performance as a measure of the effectiveness of a biomarker detection algorithm. In addition to ignoring the reproducibility performance, relying solely on the classification performance may be misleading for several reasons. First, the classification performance depends on factors other than the quality of the selected variables (i.e., biomarkers). In particular, the preprocessing steps and classifier model employed significantly impact the classification performance. Second, in the small sample size setups, the empirical estimation of classification accuracy may not reflect the true performance of a classifier.

Surprisingly, the existing state-of-art metagenomic biomarker detection algorithms have not considered the reproducibility performance in their assessment. This calls the utility of these methods under question. Similarly, assessing a biomarker detection algorithm based on its stability performance is delusive. For example, a trivial algorithm that returns the same features irrespective of the training samples will achieve a perfect stability performance. Thus, reproducibility needs to be assessed together with the classification performance.

### Simulation setup

The classification and consistency metrics were used to measure the efficiency of the six biomarker detection algorithms in identifying potential markers. The consistency-classification evaluation protocol is shown in Algorithm 2. In our experiments, a random subsampling without replacement is utilized to generate 500 subsamples (i.e., *K*=500) variations of the original dataset. Each subsample contains 80% of the samples in the original dataset (i.e., *r*=0.8). The classification and consistency performance were evaluated at different number of selected markers to provide further insights on the performance of the competing algorithms under varying sizes of the biomarker sets. The reported results represent the average over the 500 experiments.

### Dogs with exocrine pancreatic insufficiency (EPI) dataset

**Fig. 1:**
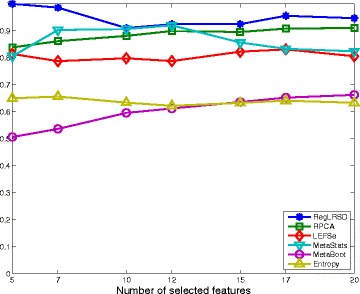
Average of Kuncheva Index (KI) at varying number of selected markers for the six biomarker detection algorithms over the dogs with EPI dataset

The reproducibility performance in terms of the average KI stability values over all the pairwise comparisons (i.e., *K*(*K*−1)/2=124750 comparisons; *K*=500) of the six algorithms for a changing number of biomarkers from the EPI dataset is illustrated in Fig. 1. As it is transparent from Fig. 1, RegLRSD outperforms all the other algorithms. The improvement gain of RegLRSD over the other algorithms in terms of reproducibility performance is higher at lower number of selected markers. This indicates that RegLRSD is more certain in identifying small subsets of potential markers.

**Fig. 2:**
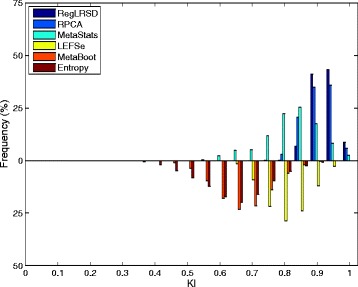
Histogram plots of the KI values generated by the six biomarker detection algorithms over the dogs with EPI dataset. Each histogram is created using 124750 values of KI which are generated from all pairwise comparisons over the *K*=500 runs (i.e., $\tfrac {K(K-1)}{2}= 124750$ comparisons)

Figure 2 presents the histogram of the KI index computed over the 124750 pairwise comparisons when the size of the selected biomarkers equals 20. The concentration of the histogram of RegLRSD at high KI values reveals that the RegLRSD algorithm achieves a high reproducibility performance. In particular, RegLRSD provides a stability value that is larger than or equal to 90% for almost 90% of the times. On the other hand, the other algorithms are less prone to achieve the same stability performance. In particular, RPCA, LEFSe, and MetaStats yield a stability performance that is larger than or equal to 90% for only 75, 15, and 30% of the times, respectively, and less than 5% of the times for both MetaBoot, and entropy-based algorithm. Moreover, the spread of the histograms of LEFSe, MetaStats, MetaBoot and entropy algorithms over wide range of KI values indicates a serious inconsistency problem that puts the outcomes of these algorithms under question.

**Fig. 3:**
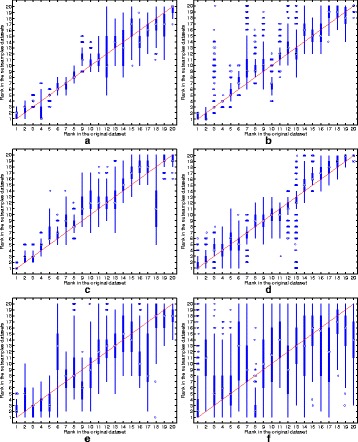
Rank boxplots in the subsamples against rank in the original data set for the six algorithms over the dogs with EPI dataset. **a** RegLRSD. **b** RPCA. **c** LEFSe. **d** MetaStats. **e** MetaBoot. **f** Entropy

The ranking stability of the selected microbial signatures over all the *K*=500 variations of the original dataset is depicted in Fig. 3. In addition to the high reproducibility performance, the RegLRSD algorithm corroborates its ability to preserve the order (i.e., rank) of the selected markers as revealed from the concentration of the boxplots of the ranks around the 45° line. The spread of the rank boxplots of the other algorithms indicates that the rank of the selected markers in these algorithms varies significantly with respect to small variations in the dataset. For example, the rank of the marker that is ranked sixth when applying the MetaBoot algorithm over the original dataset varies significantly over 500 different subsamples as cleared from Fig. 3.e. Specifically, the median value for all these ranks (i.e., ranks obtained in the 500 subsamples) equals 13 and the interquartile range (IQR) equals 6 (from 9 to 15). Moreover, in some subsamples, this marker was ranked first, while in other subsamples it was ranked twentieth.

**Fig. 4:**
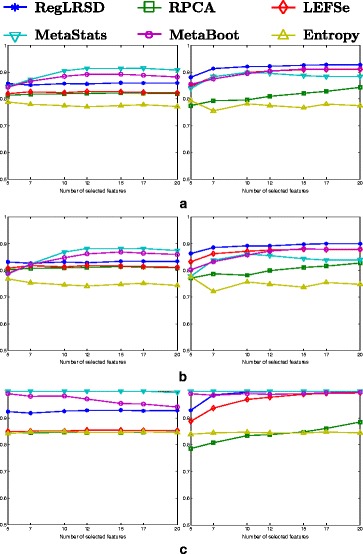
Classification performance of the six biomarker detection algorithms using the two classification algorithms, NCC-1 (column 1) and NCC-2 (column 2), over the dogs with EPI dataset. Classification performance is measured in terms of **a** accuracy, **b** sensitivity and **c** specificity

The classification performance of the competing algorithms is illustrated in Fig. 4. The first column in Fig. 4 depicts the outcomes for the NCC-1 classifier, while the second column illustrates the outcomes for the NCC-2 classifier. In general, all the algorithms yield a robust performance regardless of the number of selected biomarkers. The identified markers by RegLRSD, LEFSe, MetaStats, and MetaBoot show high ability to distinguish between healthy and diseased samples related to EPI as revealed by the high accuracy, sensitivity and specificity of these algorithms compared to RPCA and entropy algorithms, especially when the NCC-2 is used. The better performance of RegLRSD compared to RPCA demonstrates that incorporating the prior knowledge improves the performance markedly.

**Fig. 5:**
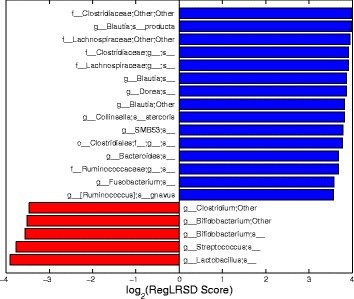
Biomarker discovery results when we selected the top 20 markers from the dogs with EPI dataset using the RegLRSD algorithm. *Blue* and *red* bars represent microbes that are enriched in control and diseased samples, respectively

Figure 5 displays the top 20 identified markers by RegLRSD and their scores. RegLRSD suggests that the EPI may be characterized by the decrease in Blautia, Bacteroides, Fusobacterium, Ruminococcus genera in dogs with EPI. Also, the genera, Lactobacillus, Streptococcus, Bifidobacterium present a significant growth in their abundance levels in dogs with EPI when compared to healthy dogs. Previous studies have also showed an increase in Lactobacillus and Streptococcus abundance levels in dogs with EPI. In particular, two culture-based investigations reported an increased number of Lactobacillus and Streptococcus in the duodenum [43], jejunum and colon of dogs with EPI [44].

### Dogs with idiopathic inflammatory bowel disease (IBD) dataset

**Fig. 6:**
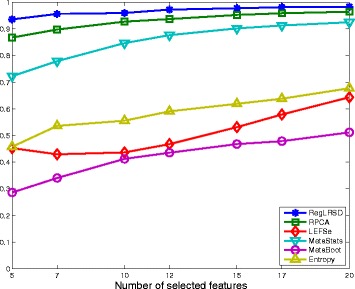
Average of Kuncheva Index (KI) at varying number of selected markers for the six biomarker detection algorithms over the dogs with IBD dataset

The stability performance measured in terms of the average KI values for the six algorithms over different numbers of biomarkers is depicted in Fig. 6. The results in Fig. 6 illustrate that RegLRSD outperforms the rest of the algorithms in terms of reproducibility performance. Moreover, adding the smoothing constraint in RegLRSD results in an improvement in the stability performance by almost 2−7*%* over the standard RPCA. Noticeably, LEFSe and MetaBoot provide a poor reproducibility performance. For example, the average KI values range around 30*%*−50*%* for MetaBoot and around 40*%*−65*%* for LEFSe.

**Fig. 7:**
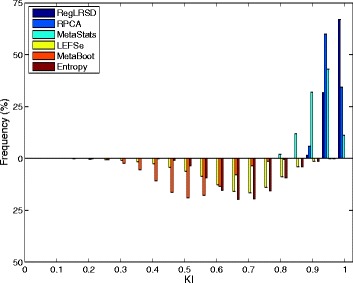
Histogram plots of the KI values generated by the six biomarker detection algorithms over the dogs with IBD dataset. Each histogram is created using 124750 values of KI which are generated from all pairwise comparisons over the *K*=500 runs (i.e., $\tfrac {K(K-1)}{2}= 124750$ comparisons)

The histograms of the KI index computed over the 124750 pairwise comparisons when the size of the selected biomarkers equals 20 is depicted in Fig. 7. The histogram of RegLRSD illustrates the superior performance of RegLRSD as it achieves 100% stability for more than 65% of the times. RPCA and MetaStats show an adequate consistency. On the other hand, LEFSe, MetaBoot, and entropy-based approach tend to provide poor performance as their corresponding histograms are centered at low KI values and spread over wide range of KI values.

**Fig. 8:**
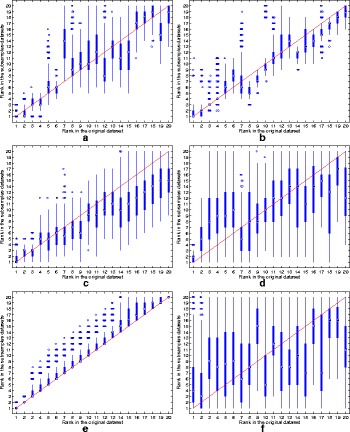
Rank boxplots in the subsamples against rank in the original data set for the six algorithms over the dogs with IBD dataset. **a** RegLRSD. **b** RPCA. **c** LEFSe. **d** MetaStats. **e** MetaBoot. **f** Entropy

**Fig. 9:**
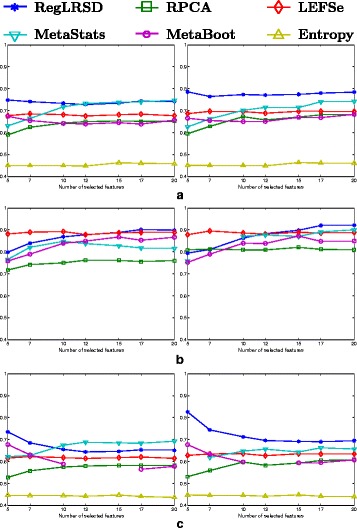
Classification performance of the six biomarker detection algorithms using the two classification algorithms, NCC-1 (column 1) and NCC-2 (column 2), over the dogs with IBD dataset. Classification performance is measured in terms of **a** accuracy, **b** sensitivity and **c** specificity

The ranking stability of the selected microbial signatures over all the *K*=500 subsamples is presented in Fig. 8. The rank of the selected markers by RegLRSD, RPCA, and MetaBoot is more consistent against the variation in the dataset. This contrasts the performance of the LEFSe, MetaStats, and entropy-based algorithms, in which the importance (i.e., rank) of the selected features varies drastically due to adding/removing a small number of samples from the original dataset. In terms of classification performance, the RegLRSD algorithm outperforms the other algorithms especially when the NCC-2 classifier is used as revealed from Fig. 9. Noticeably, RegLRSD yields a significant improvement over the RPCA algorithm. This reflects the efficiency of incorporating the prior knowledge information in generating more accurate results.

**Fig. 10:**
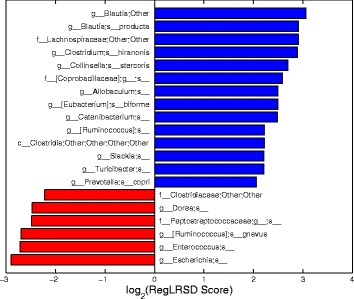
Biomarker discovery results when we selected the top 20 markers from the dogs with IBD dataset using the RegLRSD algorithm. *Blue* and *red* bars represent microbes that are enriched in control and diseased samples, respectively

RegLRSD suggested several bacterial groups as potential markers for IBD. The top 20 detected biomarkers by the RegLRSD algorithm and their scores are displayed in Fig. 10. At higher phylogenetic levels, the majority of these bacterial groups belong to Firmicutes, Bacteroidetes, and Proteobacteria. In particular, the Enterobacteriaceae is the main driver for increasing the abundance level of Gammaproteobacteria in dogs with IBD. The quantitative PCR (qPCR) assays suggest that this increase is mainly due to Escherichia coli (i.e., E. coli). Several studies in human patients with IBD [45, 46] reported that E. Coli exhibits virulent potential such as adhesive capacity, invasive capacity, toxin production, and inflammatory cytokine stimulation. Similarly, the results in [47] associated several adherent and invasive strains of E. Coli with granulomatous colitis in boxer dogs. RegLRSD have suggested several genera belonging to Firmicutes to be as a potential markers for IBD. In particular, Blautia, Turicibacter, and Faecalibacterium were decreased in IBD. Most of these bacterial groups belong to Clostridium clusters IV and XIVa and are recognized as the major producer of several metabolites including short chain fatty acids (SCFA). Consequently, decreasing the abundance level of these bacterial groups may impact the host health. These findings comply with previous studies in duodenal mucosal/luminal content and feces in dogs with IBD [48–50].

### Mouse model of ulcerative colitis (UC) dataset

**Fig. 11:**
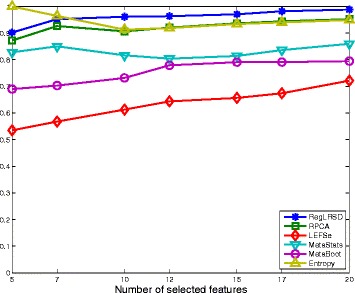
Average of Kuncheva Index (KI) at varying number of selected markers for the six biomarker detection algorithms over the mouse model of UC dataset

**Fig. 12:**
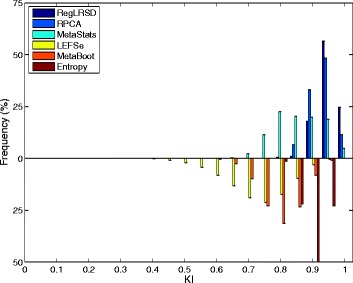
Histogram plots of the KI values generated by the six biomarker detection algorithms over the mouse model of UC dataset. Each histogram is created using 124750 values of KI which are generated from all pairwise comparisons over the *K*=500 runs (i.e., $\tfrac {K(K-1)}{2}= 124750$ comparisons)

The mean KI values across all the pairwise comparisons and their histograms in the presence of the 20 selected biomarkers are presented in Figs. 11 and 12, respectively. Figure 11 demonstrates that RegLRSD outperforms all the other algorithms and exhibits a high reproducibility performance. In particular, the improvement gain is about 5% over RPCA and entropy-based algorithm, 15% over MetaStats, 20−25*%* over MetaBoot, and more than 30% over LEFSe.

**Fig. 13:**
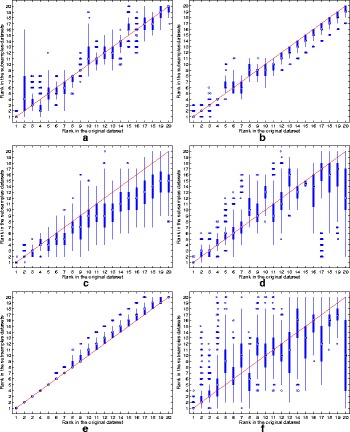
Rank boxplots in the subsamples against the rank in the original data set for the six algorithms over the mouse model of UC dataset. **a** RegLRSD. **b** RPCA. **c** LEFSe. **d** MetaStats. **e** MetaBoot. **f** Entropy

The ranking stability of the selected microbial signatures over all the *K*=500 subsamples is illustrated in Fig. 13. The outcomes in Fig. 13 point a serious inconsistency problem in the performance of LEFSe, MetaStats and entropy-based algorithm. The two matrix decomposition-based algorithms (i.e., RegLRSD and RPCA) provide a comparable performance in terms of retaining the rank of the selected markers over different subsamples of the data set.

**Fig. 14:**
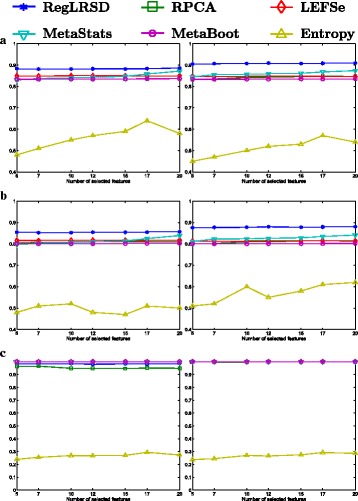
Classification performance of the six biomarker detection algorithms using the two classification algorithms, NCC-1 (column 1) and NCC-2 (column 2), over the mouse model of UC dataset. Classification performance is measured in terms of **a** accuracy, **b** sensitivity and **c** specificity

The classification performance of the six algorithms in the presence of a changing number of biomarkers from the UC mice model data set is illustrated in Fig. 14. The results in Fig. 14 point out that all the algorithms, except the entropy-based algorithm, provide almost the same classification accuracy (i.e., 80−84*%*).

**Fig. 15:**
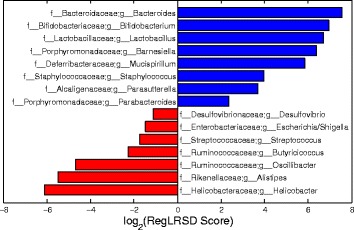
Biomarker discovery results when we selected the top 15 markers from the mouse model of UC dataset using the RegLRSD algorithm. *Blue* and *red* bars represent microbes that are enriched in control and diseased samples, respectively

The top 15 detected biomarkers by the RegLRSD algorithm are depicted in Fig. 15. The majority of these markers comply with the previous studies. For example, the authors of [51, 52] reported reduced concentrations of Lactobacillus and Bifidobacterium in colonic biopsy specimens in patients with active UC. The study [53] has suggested that the UC could be depicted via a decline in the abundance levels of Bacteroides. The authors of [9] reported that the decrease in the abundance levels of acetate producer clades such as Ruminococcaceae may reduce the host capability to fix the epithelium and to regulate inflammation. This may explain the selection of Oscillibacter, which belongs to Ruminococcaceae, as possible marker for UC. Subjects with UC showed significant reduction in Helicobacter pylori [54], the most well-known known species of Helicobacter genus.

## Conclusions

Recent advancements in metagenomic sequencing associated microbes with several health and disease states of the host. Identifying potential metagenomic markers is essential for understanding biological systems and designing possible therapies for diseases. Therefore, developing robust and stable biomarker detection algorithms is crucial in order to infer correct biological statements and translate these results into clinical practice. Herein paper, we developed the RegLRSD algorithm for biomarker detection. Apart from the conventional statistical and feature selection frameworks to tackle the problem of finding potential metagenomic biomarkers, RegLRSD formulates the biomarker detection as a matrix decomposition problem. In particular, RegLRSD models the abundance profiles of relevant and irrelevant microbes as sparse and low-rank matrices, respectively. This renders identifying potential biomarkers as the problem of decomposing the bacterial abundance data matrix into a sparse matrix and a low-rank matrix.

To enhance the accuracy of estimating the low-rank matrix and the sparse matrix, RegLRSD constrains the low rank matrix to be smooth in order to integrate the prior knowledge that the abundance profiles of irrelevant bacteria do not exhibit strong variation between different phenotypes in the biomarker detection process. Then we developed an efficient solution for this decomposition problem by exploting the alternating direction method of multipliers. In addition to the computationally efficient solution for RegLRSD, a major advantage of RegLRSD is the convex formulation of the biomarker detection problem. This convex formulation enables adding convex constraints that reflect our prior knowledge about the biological system under study. These additional constraints help in designing better algorithms that are more accurate and provide more consistent biological findings. The improved performance of RegLRSD over the conventional RPCA algorithm (i.e., without the smoothness constraint) demonstrates the efficiency of incorporating prior knowledge in the design of a biomarker detection algorithm.

In addition to the development of a novel algorithm for identifying metagenomic markers (i.e., RegLRSD), this paper addressed an important feature of the metagenomic biomarker discovery algorithms. This feature is the ability of biomarker detection algorithms to generate reproducible results. This is crucial to translate the outcome of these algorithms into practical applications. Surprisingly, the stability/reproducibility performance was not addressed by the existing metagenomic biomarker identification algorithms. Our simulation results demonstrate that the existing methods for metagenomic biomarker discovery present poor reproducibility performance. In particular, the spread of the histograms of LEFSe, MetaStats, MetaBoot and entropy-based algorithm over a wide range of KI values indicates a serious inconsistency problem that puts the outcomes of these algorithms under question.

Comprehensive comparisons with the latest biomarker detection approaches were conducted. In particular, RegLRSD was contrasted with two statistical-based approaches (i.e., LEFSe and MetaStats), two machine learning-based algorithms (MetaBoot and entropy) and a reduced from of RegLRSD in which the smoothness constraint is not considered (i.e., RPCA). The competing algorithms were tested against three realistic metagenomic datasets. The first and second datasets pertain to healthy dogs and dogs diagnosed with EPI and IBD, respectively. The third dataset refers to a mouse model of UC. These approaches were assessed in terms of classification accuracy and reproducibility performance. The simulation results show that the detected markers by RegLRSD enable discriminating metagenomic samples belonging to different phenotypes with a quite high accuracy. Moreover, RegLRSD exhibits superior consistency performance when compared to other algorithms. This renders the RegLRSD algorithm as a robust and reliable tool to identify potential metagenomic markers that may characterize the difference between samples belonging to different phenotypes.

The results presented in this paper demonstrate that the two matrix decomposition-based algorithms (i.e., RegLRSD and RPCA) are successful in providing high reproducibility and classification accuracy performance compared to the conventional statistical and machine learning-based algorithms. This validates the idea of modeling the bacterial abundance data matrix as the superposition of a low-rank matrix representing the uninformative microbes and a sparse matrix containing the abundances of informative microbes. Moreover, the improvement in the performance of RegLRSD compared to RPCA demonstrates (i) the validity of our assumption that the abundance profiles of irrelevant bacteria are smooth, and (ii) incorporating prior knowledge in the design of a biomarker detection algorithm may lead to more robust results.

Due to the necessity of developing user-friendly tools that enable the researchers to analyze metagenomic data, RegLRSD is implemented as a standalone executable software package and is made available at https://sites.google.com/a/tamu.edu/mustafa/software/reglrsd.

## Abbreviations

ADMM: Alternating direction method of multipliers
EPI: Exocrine pancreatic insufficiency
IBD: Inflammatory bowel disease
KI: Kuncheva index
LDA: Linear discriminant analysis
NCBI: National center for biotechnology information NCC: Nearest centroid classifier
OTU: Operational taxonomic unit
PCA: Principal component analysis
PCP: Principal component pursuit
PLS: Partial least square
QIIME: Quantitative insights into microbial ecology
RDP: Ribosomal database project
RegLRSD: Regularized low rank-sparse decomposition
RPCA: Robust principal component analysis
UC: Ulcerative colitis
